# A Novel Chiral Molecularly Imprinted Electrochemical Sensor Based on β-CD Functionalized Graphene Quantum Dots for Enantioselective Detection of D-Carnitine

**DOI:** 10.3390/foods14091648

**Published:** 2025-05-07

**Authors:** Feng Yang, Xin Qi, Yan Chen, Kai Tang, Mengyang Fang, Yanwei Song, Jiufen Liu, Lianming Zhang

**Affiliations:** 1 Haikou Key Laboratory of Marine Contaminants Monitoring Innovation and Application, Haikou Marine Geological Survey Center, China Geological Survey, Haikou 571127, China; 2 Command Center for Natural Resources Comprehensive Survey, China Geological Survey, Beijing 100055, China; 3 College of Chemical and Bioengineering, Guilin University of Technology, Guilin 541004, China

**Keywords:** chirality, sensor, molecular imprinting, graphene quantum dots, D-carnitine

## Abstract

In this study, β-cyclodextrin (β-CD) functionalized graphene quantum dots (GQDs) was employed to augment the array of chiral recognition sites, thereby enhancing the affinity of GQDs/β-CD composite for imprinting molecules and realizing heightened chiral selectivity. The incorporation of GQDs/β-CD into the synthesis of molecularly imprinted polymers (MIPs), synergizing with the host-guest inclusion properties of β-CD and the abundant carboxyl groups of GQDs, enhanced the chiral recognition capacity of MIPs materials. Consequently, a novel MIPs/(GQDs/β-CD) sensor with chiral recognition capabilities tailored for D-carnitine was successfully fabricated. The binding mechanism between GQDs/β-CD and D-carnitine was elucidated via Ultraviolet-visible spectroscopy and Fourier transform infrared spectroscopy. The variation in the response signal (ΔI) of the probe molecule exhibited a linear correlation with the logarithm of D-carnitine concentration (lgC) in the range of 1.0 × 10^−12^ mol/L to 1.0 × 10^−9^ mol/L, and the detection limit (3δ/S) was calculated as 2.35 × 10^−13^ mol/L. These results underscore a 7.15-fold enhancement in the selectivity of MIPs/(GQDs/β-CD) sensor for D-carnitine recognition. Moreover, the sensor presented commendable efficacy in real-world scenarios, yielding recovery rates ranging from 98.5% to 103.0% during the determination of D-carnitine content in real samples.

## 1. Introduction

L-carnitine (β-hydroxy-γ-N-trimethyl aminobutyric acid), as a nutritional fortification agent, is widely used in foods and medicine production [1,2,3,4]. D-carnitine is a chiral isomer of L-carnitine, which has been shown to be harmful to humans, causing muscle soreness and severe muscle atrophy [5,6,7]. At present, L-carnitine produced by chemical synthesis method and D-carnitine reuse method both contain D-carnitine, among which chemical synthesis has become the main preparation method of L-carnitine because of its low cost and high efficiency [8]. In many foods, such as infant milk powder, slimming products, coffee, L-carnitine has become a commonly added nutritional booster. Hence, the establishment of D-carnitine detection and analysis method is of great significance to ensure the quality and safety of the foods [9]. Current methods for D-carnitine detection include chiral high-performance liquid chromatography [10], ion mobility spectrometry, and biosensors [11]. However, these detection methods are not only expensive and operationally complex but also require specialized personnel. With the rapid development of the food industry, it has become increasingly crucial to propose programmed monitoring technologies with easy operation, high sensitivity and selectivity.

Molecularly imprinted polymers (MIPs) have emerged as a pivotal technique for the isolation, identification, and quantification of analytes, finding extensive utility across diverse domains including food safety, environmental surveillance, life sciences, pharmaceuticals, and biomedical research [12]. MIPs-based electrochemical sensors inherited the advantages of conventional electrochemical sensors, in terms of being easy to use, fast, sensitive, low-cost, low limit of detection and ideal to be further tailored for various monitoring applications. For example, Banan et al. [13] synthesized Fe_3_O_4_ coated with SiO_2_, and subsequently acetonitrile and amiodarone were polymerized on the Fe_3_O_4_ @SiO_2_ surface to form a molecularly imprinted polymer layer. Then a molecularly imprinted electrochemical sensor was fabricated and evaluated to detect amiodarone with desirable traits such as linearity, reproducibility, precision, and accuracy. Wang et al. [14] constructed a MIPs/rGO/Ti_3_C_2_T_x_/GCE electrochemical sensor platform toward the detection of acetaminophen with low detection limit (LOD = 1.58 nM, S/N = 3), wide detection range (10^−8^–10^−3^ M), desirable selectivity and stability with good repeatability and anti-interference. In another report, ionic liquid modified poly (3,4- ethylendioxythiophene)/titanium carbide was used as a substrate material to improve detection sensitivity. MIPs film for specific recognition of L-Trp was fabricated on the surface of modified electrodes using electrochemical polymerization. The fabricated sensor exhibited good linearity ranges (10^−6^–0.1 μM and 0.1–100 μM) with a low detection limit of 2.09 × 10^−7^ μM [15]. Nevertheless, the traditional MIPs-based electrochemical sensors encounter the bottle-neck due to the low binding efficiency, poor site accessibility, low selectivity and slow binding kinetics. To address these issues, some nanomaterials are usually used as imprinting support materials to modify the electrode and further to enhance the detection performance of the proposed sensor.

β-cyclodextrin (β-CD) constitutes a class of oligosaccharides comprised of seven glucose units, distinguished by a hydrophobic cavity and an array of hydrophilic hydroxyl groups adorning its exterior surface [16]. Its exceptional molecular spatial recognition attributes confer upon remarkable chiral recognition capabilities, rendering it a staple in the realm of enantiomeric recognition and detection. Therefore, CD and its derivatives have been successfully employed as a promising co-functional monomer in the design and construction of MIPs to create high affinity recognition sites hence improving the selectivity of the resulting MIP [17]. Graphene quantum dots (GQDs) represent an innovative zero-dimensional nanomaterial synthesized through the meticulous interplay of high-temperature treatment and chemical modification of graphene [18]. Diverging from conventional graphene materials, GQDs boast diminutive particle dimensions, augmented surface areas, and unparalleled physical, chemical, optical, and electrochemical characteristics [19]. Their expansive surface area, coupled with remarkable electrochemical attributes and an abundance of carboxyl groups, endows them with immense promise for electrochemical sensor applications. Leveraging their exceptional conductivity, surface area, and profusion of carboxyl groups, GQDs stand poised to enhance the sensitivity and detection thresholds of electrochemical sensors [20].

**Scheme 1:**
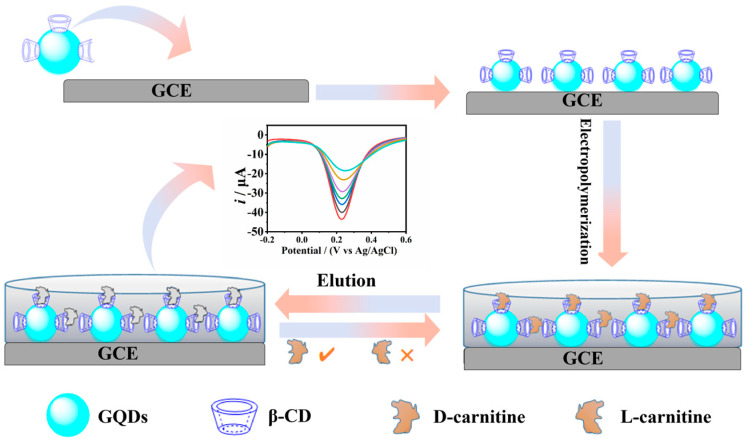
Schematic representation of the preparation for D-carnitine molecular imprinting sensor based on GQDs/β-CD.

Based on the discussion above, GQDs/β-CD composite material was introduced as an auxiliary recognition element in the preparation of MIPs through hydrogen bond binding. The negative charge resulting from the carboxyl groups of GQDs enables effective immobilization of GQDs/β-CD composite material on the surface of a glassy carbon electrode. The excellent electron transfer properties of GQDs contribute to lower detection limits in the resulting sensor. Simultaneously, β-CD, possessing chiral recognition capabilities, is introduced to enhance the chiral recognition ability of MIPs by utilizing the host-guest recognition mechanism of its cavity structure. GQDs/β-CD composite material is drop-coated onto a polished and cleaned glassy carbon electrode surface, with D-carnitine as the template molecule, to form MIPs/(GQDs/β-CD) film. After elution to remove the imprint molecule D-carnitine, specific recognition cavities for D-carnitine are formed. The mechanism of D-carnitine recognition by the sensor is illustrated in Scheme 1.

## 2. Materials and Methods

### 2.1. Reagents and Instruments

Citric acid (CA), β-cyclodextrin (β-CD), D-carnitine, L-carnitine, and o-phenylenediamine (o-PD) were procured from Shanghai Aladdin Reagent Co., Ltd. (Shanghai, China), ensuring analytical reagent (AR) purity. Potassium ferricyanide (Ⅲ), potassium ferrocyanide (Ⅱ) trihydrate, sodium dihydrogen phosphate dihydrate, disodium hydrogen phosphate dodecahydrate, ethanol, acetic acid, potassium chloride and sodium hydroxide were all sourced from Henan Huawen Chemical Co., Ltd. (Xinxiang, China). L-Threonine, L-Serine and L-Valine, distinguished by their molecular formulas and AR purity, were obtained from Hebei Bailinwei Superfine Materials Co., Ltd. (Handan, China). Additionally, D-Threonine, D-Serine, and D-Valine, each characterized by their respective molecular formulas and AR purity, were procured from Beijing Yita Biotechnology Co., Ltd. (Beijing, China).

The CHI760E electrochemical workstation, crafted by Shanghai Chenhua Instruments Co., Ltd. (Shanghai, China), served as the primary apparatus for investigating electrochemical behaviors. Fourier transform infrared (FT-IR) spectroscopy was conducted using the NEXUS 470 Infrared Spectrophotometer, manufactured by Thermo Fisher Scientific Inc. (Waltham, MA, USA). The UV-Visible spectra were obtained by UV-2400PC (Shimadzu, Kyoto, Japan) spectrophotometer with a scanning interval of 0.5 nm and a scanning range of 190.0 nm to 400.0 nm. Scanning electron microscopy was carried out by using a SU5000 microscope (JEOL, Akishima, Japan) with an acceleration voltage of 5.0 kV and a magnification of 40.0 kV. Transmission electron microscopy (TEM) was performed using a JEM-2010F microscope (JEOL, Japan) operated at 200 kV. X-ray photoelectron spectroscopy (XPS) was carried out on an AXIS HIS 165 spectrophotometer (Kratos Analytical, Manchester, UK). X-ray diffraction (XRD) experiments were tested on a D/Max-2500 V X-ray diffractometer (Rigaku, Tokyo, Japan) with Cu-Kα_1_ radiation. High-temperature material processing was facilitated by the Muffle Furnace (KSL-1100-S) engineered by Hefei Kejing Materials Technology Co., Ltd. (Hefei, China).

### 2.2. Preparation of GQDs and GQDs/β-CD

GQDs was prepared based on the method reported in reference [21] by using citric acid as a precursor with some modifications. In a crucible, 0.5 g of citric acid was sintered at 200 °C in a muffle furnace for 30.0 min. Upon the culmination of the reaction, a brownish-yellow substance was acquired. After cooling to ambient temperature, 15.0 mL of 1.0 mg/mL NaOH was uniformly introduced into the aboved substance. The resultant solution was centrifuged at 3500 rpm for 20 min, and the supernatant was then quantified and dissolved in ultrapure water to get a 2.0 mg/mL solution, which was thereafter preserved at −4 °C for subsequent utilization.

For the modification of β-CD, 0.1135 g was meticulously weighed and solubilized in ultrapure water to obtain a 1.0 × 10^−3^ mol/L solution. This solution was then preserved at −4 °C for future utilization. To prepare the GQDs/β-CD composite dispersion, 5.0 mL each of GQDs and β-CD solution was transferred to a 50.0 mL volumetric flask and subsequently filled to the brim. The resulting GQDs/β-CD composite dispersion was stored at −4 °C for subsequent applications.

### 2.3. Electrochemical Measurements

All electrochemical measurements were conducted on CHI 760E electrochemical workstation with a conventional three-electrode system, including a modified glassy carbon electrode (GCE, diameter of 3 mm) as the working electrode, a platinum wire as the counter electrode and a Ag/AgCl as the reference electrode. A solution of 5.0 × 10^−3^ mol/L [Fe(CN)_6_]^3−/4−^ containing 0.1 mol/L KCl was used as the probe molecule. The following parameters were employed for different electrochemical techniques: Cyclic voltammetry (CV) with a scan rate of 0.05 V/s and a scan potential range from −0.2 V to +0.6 V, Differential pulse voltammetry (DPV) with a scan rate of 0.05 V/s and a potential range from −0.2 V to +0.6 V, CV Electro-polymerization method with a scan rate of 0.05 V/s and a scan potential range from 0 V to +0.8 V. Electrochemical impedance spectroscopy (EIS) was conducted with an alternating amplitude of 0.05 V and a frequency range from 100.0 kHz to 0.1 Hz.

### 2.4. Sensors Fabrication

The GCE was polished with 0.05 μm Al_2_O_3_ powder on a velvet surface, followed by ultrasonic treating and meticulous cleansing with ultrapure water to expunge impurities. Subsequent air-drying ensued, followed by drop-coating 6.0 μL 4 mg/L of the GQDs/β-CD composite suspension onto the surface of the GCE. After a natural drying of 30 min, 1 μL wt. 0.5% Nafion solution was dropped on the GCE surface followed by another 30 min natural dry, then the GQDs/β-CD modified electrode was acquired. Thereafter, the modified electrode was immersed in a polymerization solution containing 1.0 × 10^−4^ mol/L D-carnitine and 4.0 × 10^−4^ mol/L o-PD, wherein electro-polymerization was operated for 20 cycles to form a MIPs/(GQDs/β-CD) film on the electrode surface, thereby the MIPs/(GQDs/β-CD) modified electrode was fabricated. Subsequently, the electrode was submerged in a mixture of ethanol and acetic acid (in a volume ratio of 4:1) for 120 s to elute and expel the imprint molecule D-carnitine. The resultant imprinted cavities were thus rendered available for the specific recognition of D-carnitine, thereby acquiring the MIPs/(GQDs/β-CD) sensor tailored for specific D-carnitine recognition.

Under identical experimental parameters, the NIPs/(GQDs/β-CD) sensor was fabricated (omitting the addition of D-carnitine in the polymerization solution) to investigate the influence of D-carnitine on the specific recognition of D-carnitine by the MIPs/(GQDs/β-CD) sensor.

Concurrently, under the similar conditions, the MIPs/GCE sensor was fabricated excepting the drop-coating of GQDs/β-CD composite material onto the glassy carbon electrode surface. This sensor was employed to elucidate the impact of GQDs/β-CD material on the recognition of D-carnitine by the MIPs/(GQDs/β-CD) sensor.

### 2.5. Preparation of Real Sample

The sample treatment procedure adhered to the methodology delineated in reference for processing commercially available L-carnitine coffee (with an L-carnitine content of 1.0% and D-carnitine content of <0.001%). Initially, 0.1 g of L-carnitine coffee was meticulously weighed and deposited into a 10.0 mL centrifuge tube. To facilitate dissolution of the coffee, 2.0 mL of ethanol and 6.0 mL of ultrapure water were introduced into the centrifuge tube. Subsequently, the mixture was centrifuged at 3500 rpm for 30 min. Then the upper 1.0 mL of the clarified liquid was transferred to a 100 mL volumetric flask and diluted with ultrapure water to volume for subsequent analysis.

## 3. Results

### 3.1. Material Characterization

The morphologies of both GQDs and β-CD/GQDs were first scrutinized using SEM. The findings revealed that the GQDs synthesized via the low temperature sintering method manifested as 5−10 nm nanoparticles with uniform dispersion (Figure S1A). Notably, the morphology of the GQDs did not exhibit obvious aggregation subsequent to β-CD modification (Figure S1B). Also, the surface of GQDs has no obvious change after β-CD modification.

**Figure 1:**
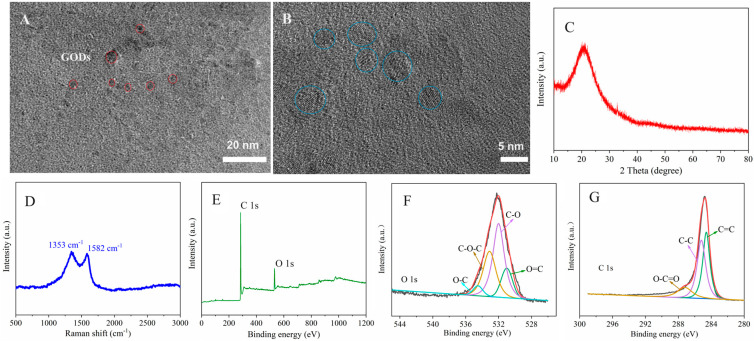
(**A**) TEM, (**B**) HRTEM, (**C**) XRD, (**D**) Raman, (**E**) full XPS spectrum, (**F**) high-resolution O 1s and (**G**) C 1s spectra of GQDs/β-CD.

The microscopic morphology of the prepared GQDs/β-CD composites was further characterized by TEM and HRTEM. Figure 1A shows that GQDs/β-CD emerged a circular shape with the even size about 5–10 nm. As displayed in Figure 1B, the lattice fringe of graphite can be observed by marking in the blue circles with the average diameters of 5 nm, which proved that the CA has been converted to GQDs during the sintering process. Figure 1C shows the XRD pattern of GQDs/β-CD with the obvious characteristic diffraction peaks of carbon materials. The peak originated at about 25°corresponding to the (002) plane of graphite, which appeared a wide and weak peak pattern because of the lower carbonization temperature. For further confirm the properties of GQDs/β-CD, the Raman spectrum of GQDs/β-CD was performed with the result displayed in Figure 1D. It appears two characteristic peaks at about 1353 cm^−1^ and 1582 cm^−1^, which can be indexed as D-band and G-band. It is generally considered that D-band represents the presence of defective carbon with the vibration of sp^3^ domains and G-band signifies the graphitic carbon layers with the vibration of sp^2^-hybridized carbon atoms. The I_D_/I_G_ value is close to 1, which reflects that GQDs/β-CD owns more defects with highly disordered carbon structure. The defects may be the active sites, which are very helpful for detecting the biomolecule by affording the adsorption or binding sites. The element compositions of the as-prepared GQDs/β-CD composite were further studied by XPS. As displayed in Figure 1E, there are two peaks at 283.2 and 531.8 eV, which corresponds to the C 1s and O 1s, respectively, where the oxygen comes from the surface oxidation of GQDs and β-CD. Figure 1F,G show the high-resolution O 1s and C 1s XPS spectra of GQDs/β-CD. For the O 1s (Figure 1F), the main characteristic peaks at 530.7, 531.5, 533.1, and 534.0 eV correspond to the O=C, C-O, C-O-C and O-C groups. The C 1s survey spectrum possesses three main characteristic peaks at 284.1, 285.8, and 290.1 eV, which represent C=C, C-C, and O-C-O groups (Figure 1G).

The investigation into the interaction among GQDs, β-CD, and D-carnitine was carried out utilizing UV-Visible spectroscopy. Spectral scans were performed within the range of 190.0 to 400.0 nm with ultrapure water serving as the background. As depicted in Figure S2, β-CD exhibited two characteristic absorption peaks located at 193.2 nm and 221.4 nm respectively, which originated from the conjugated π-electron system within the β-CD molecule. The results unveiled that the absorption peak of β-CD at 221.4 nm experienced a blue shift of 3.0 nm to 218.4 nm upon binding with GQDs, indicative of the formation of GQDs/β-CD composite facilitated by van der Waals forces and hydrogen bonding interactions [22]. Subsequent to the interaction of GQDs/β-CD with D-carnitine, the absorption peak at 218.4 nm was observed to vanish. This disappearance is attributed to the infiltration of D-carnitine into the hydrophobic cavity of the GQDs/β-CD composite, where hydrogen bonding and electrostatic interactions occur.

The interaction among GQDs, β-CD and D-carnitine were further investigated via FT-IR spectroscopy (Figure S3). In the spectrum of GQDs (curve a), distinctive absorption peaks were discerned at 3449.85 cm^−1^ (υ_OH_), 1400.59 cm^−1^ (in-plane δ_OH_), 1637.3 cm^−1^ (υ_C=O_), and 515.62 cm^−1^ (C-C=O). For β-CD (curve b), absorption peaks were evident at 3293.98 cm^−1^ (υ_OH_) and 1030.89 cm^−1^ (υ_C-O_). Subsequent to the interaction of GQDs with β-CD, the characteristic absorption peaks υC=O and C-C=O at 1637.23 cm^−1^ and 515.62 cm^−1^ underwent a shift to 1634.39 cm^−1^ and 527.36 cm^−1^, indicative of hydrogen bond formation and electrostatic interactions between GQDs and β-CD, thus corroborating the formation of the GQDs/β-CD composite material (curve c). Besides, the peak of 1030.89 cm^−1^ (υ_C-O_) originated from β-CD was not detected, which may be due to the formation of hydrogen bonds between -OH from cyclodextrins and -COOH from GQDs. In the spectrum of D-carnitine (curve d), the characteristic absorption peak at 1106.47 cm^−1^ corresponds to the υ_C-N_ of the tertiary amine group in D-carnitine. Following the recognition and binding of D-carnitine by the GQDs/β-CD composite material, the absorption peak at 1093.14 cm^−1^ may be originated from the shift of peaks at 1106.47 cm^−1^. This observation suggests that D-carnitine infiltrated the cavity of the GQDs/β-CD composite, engendering hydrogen bond formation in the process (curve e).

### 3.2. Electrochemical Characterization of Sensors

**Figure 2:**
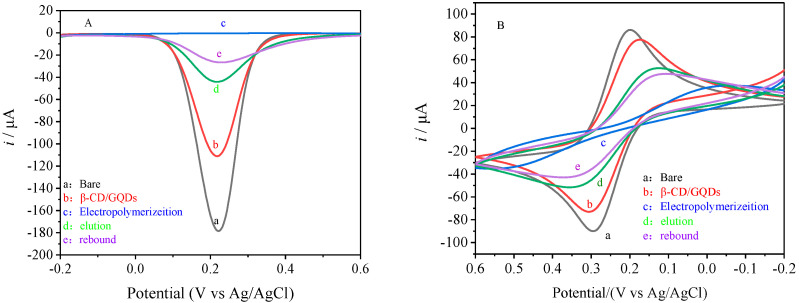
(**A**) DPV and (**B**) CV curves of MIPs/(GQDs/*β*-CD) sensor under different conditions. a: bare GCE, b: modified GQDs/*β*-CD, c: electro-polymerization, d: elution, e: rebound.

At a concentration of 5.0 × 10^−3^ mol/L [Fe(CN)_6_]^3−/4−^ containing 0.1 mol/L KCl as the probe, the electrochemical behaviors of MIPs/(GQDs/β-CD) sensor under different conditions were recorded (Figure 2A). The peak current intensity of the bare GCE was maximized at 0.2 V as determined by DPV (curve a). The drop-casting of GQDs/β-CD composite hindered electron transfer, resulting in a decrease in peak current intensity (curve b). Following electro-polymerization, a dense and poorly conductive MIPs film was formed on the electrode surface, leading to increased resistance and a DPV peak current intensity approaching zero (curve c). After washing the electrode in a 4:1 volume ratio mixture of ethanol and acetic acid for 120 s to remove D-carnitine, the current intensity increased again (curve d). This enhancement was attributed to the formation of imprinting cavities on the MIPs film, allowing electrons to transfer through the cavities and enhancing the current intensity. Subsequent re-adsorption of a 1.0 × 10^−9^ mol/L D-carnitine solution for 25 min resulted in a reduction in current intensity (curve e). This indicated that the imprinting cavities on the MIPs/(GQDs/β-CD) sensor were filled with D-carnitine, impeding electron transfer, thereby demonstrating the recognition capability of the sensor towards D-carnitine. The CV results (Figure 2B) were consistent with the DPV results.

To study the influence of physical adsorption, the electrochemical performance of NIPs/(GQDs/β-CD) sensor was scrutinized with the results depicted in Figure S4. As for DPV (Figure S4A), the peak current intensity of the bare GCE peaked at 0.2 V (curve a). After drop-casting GQDs/β-CD composite, it impeded electron transfer on the electrode surface, resulting in a decrease in current intensity (curve b). Subsequent electro-polymerization to establish a dense NIPs(GQDs/β-CD) film further impeded electron passage, reaching the lowest peak current intensity (curve c). Due to the absence of imprint molecules in the NIPs film, no discernible increase in current intensity was observed after washing the NIPs(GQDs/β-CD) sensor, verifying the absence of imprint sites (curve d). Re-adsorption of a 1.0 × 10^−9^ mol/L D-carnitine solution for 25 min emerged nearly unchanged current intensity (curve e). The results suggest that the re-adsorption of D-carnitine by the MIPs/(GQDs/β-CD) sensor does not arise from physical adsorption. The consistency between DPV (Figure S4A) and CV results (Figure S4B) offers further substantiation of the specific adsorption of D-carnitine by the MIPs/(GQDs/β-CD) sensor. Besides, the blank comparison with MIPs/GQDs formed without adding β-CD were investigated. As shown in Figure S5, the peak current intensity of the bare GCE was maximized at 0.2 V as determined by DPV (curve a). The current intensity of GQDs electrode significantly decreased because GQD hinders the electron transfer of the electrode surface (curve b). Following electro-polymerization, the electrode surface was covered by the dense MIPs film, which made the resistance increase and the DPV peak current intensity decrease to almost zero (curve c). After washing the electrode to remove D-carnitine, the peak current intensity increased slightly (curve d). Then re-adsorption of a 1.0 × 10^−9^ mol/L D-carnitine solution, the peak current intensity decreased (curve e). This confirms that the cavity structure of β-CD plays a very important role in the process of molecular recognition.

The impedance performance of the MIPs/(GQDs/β-CD) sensor was investigated using EIS with the results illustrated in Figure S6. Initially, the bare GCE exhibited the lowest charge transfer resistance value (curve a, 129.7 Ω). As GQDs/β-CD composite was drop-casted on the surface of GCE, it hindered electron transfer, further resulting in a slight increase (curve b, 380.8 Ω). The dense MIPs/(GQDs/β-CD) film originated from the subsequent electro-polymerization amplified the difficulty of electron transfer to the GCE surface, significantly elevating the resistance (curve e, 6633.1 Ω). Following the electrode’s immersion in a 4:1 volume ratio mixture of ethanol and acetic acid for 120 s to eliminate D-carnitine, imprint holes specific to D-carnitine were formed, facilitating electron transfer and reducing the impedance (curve c, 1189.3 Ω). Re-adsorption of a 1.0 × 10^−9^ mol/L D-carnitine solution for 25 min led to a re-elevation in impedance (curve e, 1379.7 Ω), indicating the capability of the prepared MIPs/(GQDs/β-CD) sensor to recognize and bind D-carnitine.

### 3.3. Optimization of Experimental Conditions

**Figure 3:**
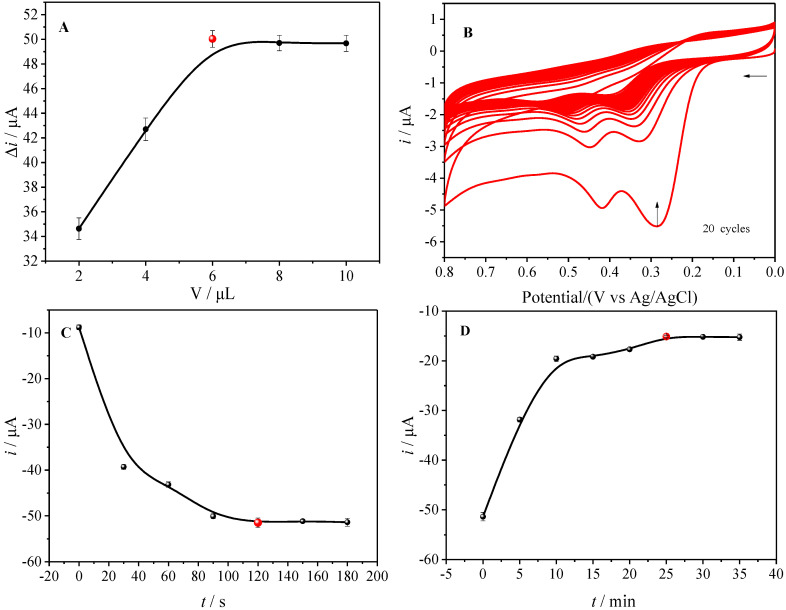
(**A**) Influence of the GQDs/β-CD drop-casting volume on the electrode surface, (**B**) Effect of electro-polymerization cycles on the current intensity; Influence of (**C**) elution time and (**D**) re-adsorption time on the current density.

The effect of the pH on the electrochemical behaviors of D-carnitine was first studied (Figure S7) in PBS solution with different pH values. It can be seen that D-carnitine emerged with the greatest electrochemical response at the pH value of 7.4 after elution and re-adsorption. When the pH was lower than 7.4, or higher than 7.4, the peak current intensity was reduced, probably because the structure of the material was affected by pH. Therefore, pH 7.4 PBS solution was chosen as the optimal electrolyte solution. The quantity of GQDs/β-CD deposited on the electrode surface plays a crucial role in determining the number of recognition sites in the MIPs/(GQDs/β-CD) sensor. To optimize the number of recognition sites, the impact of GQDs/β-CD drop-casting volume was systematically investigated. GQDs/β-CD were drop-cast onto the bare GCE in volumes ranging from 0 to 10.0 μL. As the increase of the drop-casting volume from 0 to 6.0 μL, the amount of GQDs/β-CD composite on the electrode surface gradually increased, which facilitated the electron transfer, further resulting in a continuous increase in the DPV peak current (Δi) (Figure 3A). When the drop-casting volume increased to 8.0 μL and 10.0 μL, Δi ceased to escalate further, signifying the saturation of the GQDs/β-CD composite on the sensor surface. Hence, 6.0 μL was identified as the optimal drop-casting volume for GQDs/β-CD composite material, ensuring efficient sensor performance while minimizing time investment.

In accordance with pertinent literature [23], the polymerization conditions wield substantial influence over the sensor’s sensitivity and stability. Hence, the concentrations, ratios, and the number of electro-polymerization cycles of o-PD and the imprint molecule D-carnitine were meticulously examined to optimize the sensor for heightened sensitivity and steadfastness. The findings revealed that a molar ratio of 4:1 for o-PD to D-carnitine (at a concentration of 1.0 × 10^−4^ mol/L) engendered a MIPs film endowed with exceptional stability and elution efficiency. Concurrently, the sensor demonstrated commendable re-adsorption capability for the imprint molecule D-carnitine. CV analysis unveiled a gradual decline in oxidation peak current intensity with an escalation in the number of polymerization cycles (Figure 3B). Upon reaching 20 cycles, the CV oxidation peak current intensity stabilized, indicative of the formation of a dense MIPs/(GQDs/β-CD) imprint film impeding electron transfer. Consequently, 20 electro-polymerization cycles was selected for subsequent experiments to establish a more resilient MIPs/(GQDs/β-CD) imprint film, all the while ensuring optimal elution and re-adsorption capacities for D-carnitine.

To scrutinize the influence of elution solvent type and elution time on the formation of D-carnitine imprint holes and the stability of the MIPs film, various elution solvents and times were examined to achieve an optimal sensor with heightened sensitivity and stability. Elution was executed utilizing methanol, ethanol, water-acetic acid, ethanol-acetic acid (6:1), and ethanol-acetic acid (8:1) mixtures in a volume ratio of 4:1. The findings unveiled that employing a 4:1 volume ratio ethanol-acetic acid mixture as the elution solvent yielded superior outcomes, because more imprint holes with specific adsorption for the imprint molecule D-carnitine were generated. Within the time span of 0 to 120 s, a progressive augmentation in the number of D-carnitine imprint holes and the DPV oxidation peak current (Ip) intensity ensued with escalating elution time. Following 120 s, the current intensity stabilized, prompting the selection of 120 s as the optimal elution time for subsequent experiments (Figure 3C). To explore the impact of re-adsorption time on the recognition performance of the MIPs/(GQDs/β-CD) sensor for D-carnitine, the prepared sensor was immersed in a 1.0 × 10^−9^ mol/L D-carnitine solution for re-adsorption. Within the timeframe of 0 to 25 min, a progressive filling of the imprint holes formed on the MIPs/(GQDs/β-CD) sensor surface with D-carnitine led to a gradual decline in Ip intensity. Upon reaching 25 min, the intensity stabilized, signifying that the sensor’s adsorption for D-carnitine approached saturation. Consequently, 25 min was designated as the optimal re-adsorption time for subsequent experiments (Figure 3D).

### 3.4. Analytical Performance

**Figure 4:**
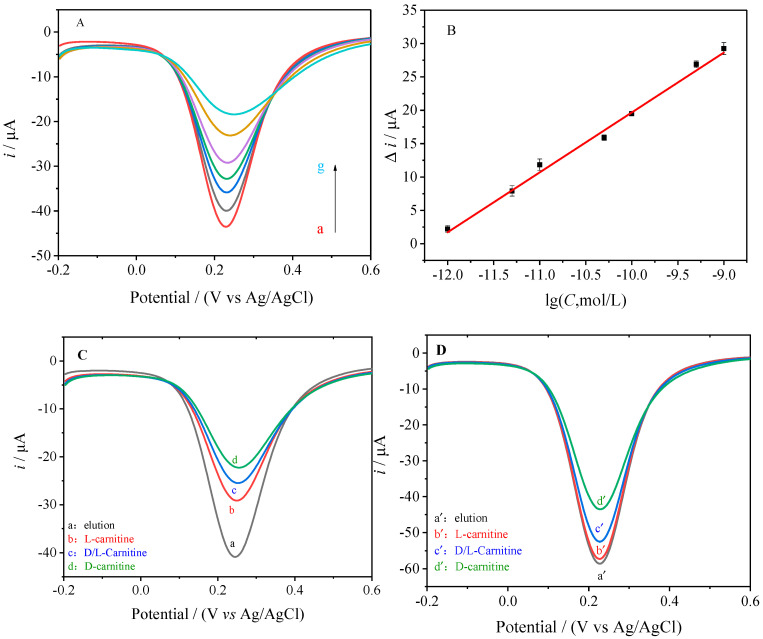
DPV signal response of the sensor to different concentrations of D-carnitine (**A**) and working curve (**B**). a→g: 1.0 × 10^−12^ mol/L, 5.0 × 10^−12^ mol/L, 1.0 × 10^−11^ mol/L, 5.0 × 10^−11^ mol/L, 1.0 × 10^−10^ mol/L, 5.0 × 10^−10^ mol/L, 1.0 × 10^−9^ mol/L of D-carnitine; Selectivity of MIPs/n sensor ((**C**), a: Elution, b: L-carnitine, c: D/L-carnitine, d: D-carnitine) and MIPs/(GQDs/β-CD) sensor ((**D**), a′: Elution, b′: L-carnitine, c′: D/L-carnitine, d′: D-carnitine).

Under the optimized working conditions, the recognition performance of the MIPs/(GQDs/β-CD) sensor for varying concentrations of D-carnitine was investigated using DPV. The peak current signal intensity of the sensor was sequentially measured in D-carnitine solutions ranging from 1.0 × 10^−12^ mol/L to 1.0 × 10^−9^ mol/L after a 25 min re-adsorption period. The results depicted in Figure 4A showcased a gradual decrease in DPV peak current with increasing concentrations of re-adsorbed D-carnitine. This decline can be attributed to the enhanced recognition of D-carnitine by the MIPs/(GQDs/β-CD) sensor, leading to the obstruction of electron transfer pathways and a consequent reduction in DPV peak current intensity.

Furthermore, the study revealed that the change in DPV response signal (Δi) of the MIPs/(GQDs/β-CD) sensor exhibited a linear relationship with the logarithm of D-carnitine concentration (lgC) within the range of 1.0 × 10^−12^ mol/L to 1.0 × 10^−9^ mol/L (Figure 4B). The linear equation was determined as Δi(A) = 8.9 × 10^−6^ lg(C/(mol/L)) + 1.1 × 10^−4^ (r = 0.9899), and the detection limit (DL, calculated as 3 times the standard deviation of the blank solution divided by the slope of the working curve) was found to be 2.35 × 10^−13^ mol/L. Upon comparison with other methods for detecting D-carnitine and their respective detection ranges and limits (Table 1), the MIPs/(GQDs/β-CD) sensor developed in this study exhibited superior sensitivity and a lower detection limit.

#### 3.4.1. Selectivity

To explore the influence of GQDs/β-CD composite material on the chiral recognition performance of the sensor, the recognition abilities of the MIPs/(GQDs/β-CD) sensor and MIPs/n sensor for carnitine enantiomers were examined. The MIPs/n sensor was evaluated for the recognition of 1.0 × 10^−9^ mol/L L-carnitine, racemic carnitine (D/L-carnitine), and D-carnitine. The results depicted in Figure 4C indicated that the MIPs/n sensor responded to L-carnitine, D/L-carnitine, and D-carnitine. The response signal intensity after recognizing L-carnitine (curve b) decreased by 28.87%, while recognizing D/L-carnitine (curve c) decreased by 37.83%, and recognizing D-carnitine (curve d) decreased by 45.83%. The MIPs/GCE sensor’s recognition ability for L-carnitine was 76.58% compared to the same concentration of D-carnitine, indicating its struggle to distinguish enantiomers with highly similar structures and sizes. Subsequently, the MIPs/(GQDs/β-CD) sensor was tested for the recognition of 1.0 × 10^−9^ mol/L L-carnitine, D/L-carnitine, and D-carnitine. The DPV results depicted in Figure 4D showed the elution peak current intensity (curve a′) as the blank control with the biggest peak current. Because cavities are formed at the electrode interface after the D-carnitine is eluted, enhancing the electron transport and resulting in the biggest current. As the L-carnitine was added, L-carnitine cannot enter the pores formed by D-carnitine, and the electrode surface hardly changes, so the peak current is almost unchanged (curve b′). As for D-carnitine, it can perfectly match with the recognition cavities on the electrode surface, resulting in the blockage of the electron transmission channels on the electrode surface and further leading to a decrease in the peak current (curve d′). When the mixture of D/L-carnitine was added, a part of the recognition cavities on the electrode surface are filled with D-carnitine, resulting in a partial decrease in current. Notably, compared to the MIPs/n sensor, the selectivity of the MIPs/(GQDs/β-CD) sensor for D-carnitine shows a significant enhancement in the sensor’s capability to recognize a single enantiomer by incorporating GQDs/β-CD composite as an auxiliary component. In addition, the DPV curves of L-carnitine at different concentrations were detected in order to further confirm whether the constructed sensor has a signal response to L-carnitine. As displayed in Figure S8, the DPV was tested in L-carnitine solutions with the concentration of 1.0 × 10^−12^ mol/L to 1.0 × 10^−9^ mol/L, it was found that the current intensity is almost constant. This disclosed that the constructed sensor can be used to detect D-carnitine without the interference from L-carnitine.

#### 3.4.2. Anti-Interference Ability

To assess the anti-interference ability of the prepared MIPs/(GQDs/β-CD) sensor, six amino acids (D-Thr, D-Ser, D-Val, L-Thr, L-Ser, L-Val), structurally similar to D-carnitine, were selected as interferents. DPV was utilized to measure the current signals of the MIPs/(GQDs/β-CD) sensor after re-adsorbing solutions containing D-carnitine (1.0 × 10^−9^ mol/L) with 1000 times the concentration (1.0 × 10^−6^ mol/L) of each interfering amino acid. The results depicted the peak current signals (Δi) after recognizing D-carnitine with the interference of the six amino acids (Figure S9). The MIPs/(GQDs/β-CD) sensor demonstrated robust anti-interference capability, with the peak current signal changes (Δi) for D-carnitine in the presence of 1000 times the concentration of interfering amino acids not exceeding 10%. The maximum interference was observed with D-Val, resulting in a signal change of 7.32% compared to the signal obtained with only D-carnitine. These results indicate that the MIPs/(GQDs/β-CD) sensor possesses excellent resistance to interference.

#### 3.4.3. Stability, Reproducibility, and Lifespan

To evaluate the stability and reproducibility of the MIPs/(GQDs/β-CD) sensor in detecting D-carnitine, one MIPs/(GQDs/β-CD) electrode was utilized to re-adsorb 1.0 × 10^−9^ mol/L D-carnitine for 25 min, the DPV response was measured nine times (Figure S10A). The relative standard deviation (RSD%) was found to be 1.91%, indicating excellent stability of the MIPs/(GQDs/β-CD) sensor. Similarly, five MIPs/(GQDs/β-CD) electrodes were prepared, and the DPV peak current intensity difference (Δi) after recognizing 1.0 × 10^−9^ mol/L D-carnitine for 25 min was measured (Figure S10B). The RSD% was 3.12%, signifying good reproducibility of the MIPs/(GQDs/β-CD) sensor. Furthermore, after storing the electrodes in a dust-proof refrigerator for 7 days, the measured DPV current response intensity only decreased by 1.69%, indicating that the prepared MIPs/(GQDs/β-CD) sensor has a relatively long lifespan.

#### 3.4.4. Real Sample Detection

To assess the practicality of the developed MIPs/(GQDs/β-CD) sensor, commercially available L-carnitine coffee was utilized as a real sample for detecting the content of D-carnitine. The MIPs/(GQDs/β-CD) sensor was immersed in the test solution for 25 min for re-adsorption, and the DPV was recorded. The detection results are presented in Table 2. In the coffee sample (1.54 × 10^−3^ g/L), the detected concentration of D-carnitine was 1.16 × 10^−11^ mol/L, which was lower than the labeled content (<0.001%). The standard addition method was employed to verify the accuracy of the MIPs/(GQDs/β-CD) sensor with a recovery rate ranging from 98.5% to 103.0%. The results suggest that the MIPs/(GQDs/β-CD) sensor can be effectively utilized for the detection of D-carnitine content in real samples.

## 4. Discussion

This study introduces a chiral recognition method utilizing GQDs/β-CD composite and establishes a sensor highly adept at D-carnitine molecules. The fabricated sensor exhibits high recognition ability for D-carnitine in optimal conditions due to the functionalization of β-CD augment the array of chiral recognition sites and the hydrophilia, thereby enhancing the affinity of GQDs/β-CD composite for imprinting molecules. The developed MIPs/(GQDs/β-CD) shows wider linear ranges (1.0 × 10^−12^ mol/L to 1.0 × 10^−9^ mol/L), lower detection limit (3δ/S, 2.35 × 10^−13^ mol/L), high selectivity, good reproducibility and acceptable stability. Besides, it offers notable benefits including ease of operation, exceptional selectivity, and efficiency in terms of time and cost. These attributes render it promising for the trace detection of chiral molecules.

## Figures and Tables

**Table 1:** Comparison of analytical parameters for D-carnitine with different analytical methods.

Detection Method	Linear Range (mol/L)	Detection Limit (mol/L)	Reference
Raman Spectroscopy	1.0 × 10^−6^~1.0 × 10^−3^	1.0 × 10^−6^	[24]
Amperometry Biosensor	4.0 × 10^−14^~6.0 × 10^−12^	2.0 × 10^−15^	[25]
Capillary Electrophoresis-Tandem Mass Spectrometry	6.2 × 10^−8^~3.7 × 10^−4^	6.2 × 10^−8^	[26]
MIPs/(GQDs/β-CD) Sensor	1.0 × 10^−12^~1.0 × 10^−9^	2.35 × 10^−13^	This work

**Table 2:** Detection of actual samples.

Sample	Found (mol/L)	RSD% n = 5	Added (mol/L)	Total Found (mol/L)	RSD% n = 5	Recovery %
L-Carnitine Coffee	1.16 × 10^−11^		1.00 × 10^−11^	2.19 × 10^−11^	3.26	103.0
		3.42	2.00 × 10^−11^	3.13 × 10^−11^	2.64	98.5
			3.00 × 10^−11^	4.21 × 10^−10^	2.37	101.7

## Data Availability

The original contributions presented in the study are included in the article; further inquiries can be directed to the corresponding authors.

## Supplementary Materials

The following supporting information can be downloaded at: https://www.mdpi.com/article/10.3390/foods14091648/s1, Figure S1: SEM images of (A) GQD and (B) β-CD/GQDs; Figure S2: UV absorption spectra of, β-CD, GQDs, GQDs/β-CD, D-carnitine and (GQDs/β-CD)+D-carnitine; Figure S3: FT-IR spectra of (A) GQDs, β-CD and GQDs/β-CD; and (B) GQDs/β-CD+D-carnitine, GQDs/β-CD and D-carnitine; Figure S4: DPV(C) and CV(D) responses of NIPs/(GQDs/β-CD) sensor under different conditions. a: bare GCE, b: modified GQDs/β-CD, c: electropolymerization, d: elution, e: rebound; Figure S5: DPV responses of MIPs/GQDs sensor under different conditions. a: bare GCE, b: modified GQDs/β-CD, c: electropolymerization, d: elution, e: rebound; Figure S6: EIS response of MIPs/(GQDs/β-CD) sensor. a: bare GCE, b: modified GQDs/β-CD, c: electropolymerization, d: elution, e: rebound; and the insert is equivalent circuit; Figure S7: DPV curves of MIPs/(GQDs/β-CD) sensor under the conditions of (A) elution and (B) rebound at the different pH values of PBS; Figure S8: DPV signal response of the sensor to different concentrations of L-carnitine. (1.0 × 10^−12^ mol/L, 5.0 × 10^−12^ mol/L, 1.0 × 10^−11^ mol/L, 5.0 × 10^−11^ mol/L, 1.0 × 10^−10^ mol/L, 5.0 × 10^−10^ mol/L, 1.0 × 10^−9^ mol/L of L-carnitine). Figure S9: Anti-interference experiment of MIPs/(GQDs/β-CD) sensor against D-Thr, D-Ser, D-Val, L-Thr, L-Ser, L-Val at 1000 times concentration; Figure S10: Determination of (A) stability and (B) reproducibility of the MIPs/(GQDs/β-CD) sensor.

## References

[1] Motazedian N., Ashari A., Dehdari Ebrahimi N., Sayadi M., Pourjafar S., Motazedian N., Khademi V., Shamsaeefar A., Eshraghian A. (2024). Effects of L-carnitine on frailty status in patients with liver cirrhosis: A randomized-controlled trial. Health Sci. Rep..

[2] Carillo M.R., Bertapelle C., Scialò F., Siervo M., Spagnuolo G., Simeone M., Peluso G., Digilio F.A. (2020). L-Carnitine in drosophila: A review. Antioxidants.

[3] Yang Y., Xue X., Zhou J., Qiu Z., Wang B., Yin Z., Ou G., Zhou Q. (2024). L-carnitine combined with traditional Chinese medicine for male infertility: A systematic review and meta-analysis. Heliyon.

[4] Gziut T., Thanacoody R. (2024). L-carnitine for valproic acid-induced toxicity. Br. J. Clin. Pharmacol..

[5] Cheng B., Li K., Li W., Liu Y., Zheng Y., Zhang Q., Chen D. (2024). Carnitine analysis in food and biological samples: Chromatography and mass spectrometry insights. Arab. J. Chem..

[6] Brittain E.L., Lindsey A., Burke K., Agrawal V., Robbins I., Pugh M., Calcutt M.W., Mallugari R., West J., Nian H. (2024). Carnitine consumption and effect of oral supplementation in human pulmonary arterial hypertension: A pilot study. Pulm. Circ..

[7] Uner B., Ergin A.D., Ansari I.A., Macit-Celebi M.S., Ansari S.A., Kahtani H.M.A. (2023). Assessing the in vitro and in vivo performance of L-carnitine-loaded nanoparticles in combating obesity. Molecules.

[8] Bagheri S., Samiee S., Zarif M.N., Deyhim M.R. (2023). l-carnitine modulates free mitochondrial DNA DAMPs and platelet storage lesions during storage of platelet concentrates. J. Thromb. Thrombolysis.

[9] Curcio R., Lunetti P., Zara V., Ferramosca A., Marra F., Fiermonte G., Cappello A.R., De Leonardis F., Capobianco L., Dolce V. (2020). Drosophila melanogaster mitochondrial carriers: Similarities and differences with the human carriers. Int. J. Mol. Sci..

[10] Liu G., Deng W., Cui W., Xie Q., Zhao G., Wu X., Dai L., Chen D., Yu B. (2020). Analysis of amino acid and acyl carnitine profiles in maternal and fetal serum from preeclampsia patients. J. Matern.-Fetal Neonatal Med..

[11] Zhang L., Luo K., Li D., Zhang Y., Zeng Y., Li J. (2020). Chiral molecular imprinted sensor for highly selective determination of D-carnitine in enantiomers via dsDNA-assisted conformation immobilization. Anal. Chim. Acta.

[12] Sarvutiene J., Prentice U., Ramanavicius S., Ramanavicius A. (2024). Molecular imprinting technology for biomedical applications. Biotechnol. Adv..

[13] Banan K., Niknam S., Ahmadi M., Tabasi S., Ghalkhani M., Adabi M., Ghorbani-Bidkoreph F. (2024). Molecularly imprinted electrochemical sensor based on carbon nanofibers for Amiodarone determination. Microchem. J..

[14] Wang Q., Liu J., Ren S., Zheng Z. (2024). Construction of molecularly imprinted voltammetric sensor based on rGO/Ti_3_C_2_T_x_ for the selective determination of acetaminophen. Microchem. J..

[15] Xue C., Jamal R., Abdiryim T., Liu X., Liu F., Xu F., Cheng Q., Tang X., Fan N. (2024). An ionic liquid-modified PEDOT/Ti_3_C_2_TX based molecularly imprinted electrochemical sensor for pico-molar sensitive detection of L-Tryptophan in milk. Food Chem..

[16] Roy A., Manna K., Dey S., Pal S. (2023). Chemical modification of β-cyclodextrin towards hydrogel formation. Carbohydr. Polym..

[17] Wen Y., Wang M., Gong W., Wang H., Fan H., Li H., Wang J., Sun B. (2024). Molecularly imprinted electrochemical sensor based on α-Cyclodextrin inclusion complex and MXene modification for highly sensitive and selective detection of alkylresorcinols in whole wheat foods. J. Agric. Food Chem..

[18] Tian P., Tang L., Teng K.S., Lau S.P. (2018). Graphene quantum dots from chemistry to applications. Mater. Today Chem..

[19] Li M., Chen T., Gooding J.J., Liu J. (2019). Review of carbon and graphene quantum dots for sensing. ACS Sens..

[20] Yan Y., Gong J., Chen J., Zeng Z., Huang W., Pu K., Liu J., Chen P. (2019). Recent advances on graphene quantum dots: From chemistry and physics to applications. Adv. Mater..

[21] Diao J., Wang T., Li L. (2019). Graphene quantum dots as nanoprobes for fluorescent detection of propofol in emulsions. R. Soc. Open Sci..

[22] Ou J., Zhu Y., Kong Y., Ma J. (2015). Graphene quantum dots/β-cyclodextrin nanocomposites: A novel electrochemical chiral interface for tryptophan isomer recognition. Electrochem. Commun..

[23] AL-Ammari R.H., Ganash A.A., Salam M.A. (2019). Electrochemical molecularly imprinted polymer based on zinc oxide/graphene/poly (o-phenylenediamine) for 4-chlorophenol detection. Synth. Met..

[24] Adeva-Andany M.M., Calvo-Castro I., Fernández-Fernández C., Donapetry-García C., Pedre-Piñeiro A.M. (2017). Significance of l-carnitine for human health. IUBMB Life.

[25] Stefan R.I., Bokretsion R.G., van Staden J.F., Aboul-Enein H.Y. (2003). Simultaneous determination of L-and D-carnitine using a sequential injection analysis/amperometric biosensors system. J. Pharm. Biomed. Anal..

[26] Sánchez-Hernández L., Castro-Puyana M., García-Ruiz C., Crego A.L., Marina M.L. (2010). Determination of L-and D-carnitine in dietary food supplements using capillary electrophoresis–tandem mass spectrometry. Food Chem..

