# Supplementary material for: A Novel Chiral Molecularly Imprinted Electrochemical Sensor Based on β-CD Functionalized Graphene Quantum Dots for Enantioselective Detection of D-Carnitine
Source: Foods. 2025 May 7;14(9):1648. doi: 10.3390/foods14091648 (PMC12071952; doi:10.3390/foods14091648)
Supplement: Supplementary file 1 [file foods-14-01648-s001.zip › foods-3574378-supplementary.pdf]

Supporting information

A Novel Chiral Molecularly Imprinted Electrochemical Sensor Based on on β-CD Functionalized Graphene Quantum Dots for Enantioselective Detection of D-Carnitine

Feng Yang [1,†], Xin Qi [1,†], Yan Chen [1], Kai Tang [1], Mengyang Fang [1], Yanwei Song [1], Jiufen Liu [2,] and Lianming Zhang [3,*]*

[1] Haikou Key Laboratory of Marine Contaminants Monitoring Innovation and Application, Haikou Marine Geological Survey Center, China Geological Survey, Haikou 571127, China

[2] Command Center for Natural Resources Comprehensive Survey, China Geological Survey, Beijing 100055, China

[3] College of Chemical and Bioengineering, Guilin University of Technology, Guilin 541004, China

* Correspondence: 13863858360@163.com (J.L.); lianming226@126.com (L.Z.)

[†] These authors contributed equally to this work.

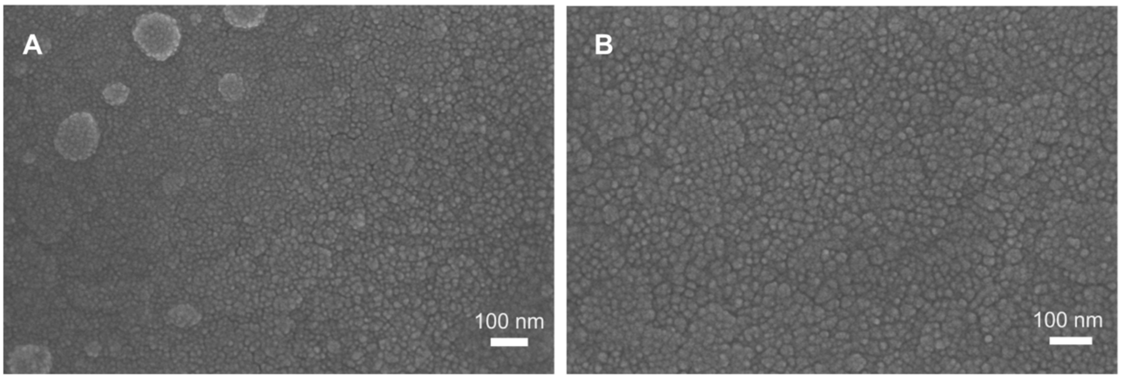

Figure S1. SEM images of (A) GQD and (B) β-CD/GQDs.

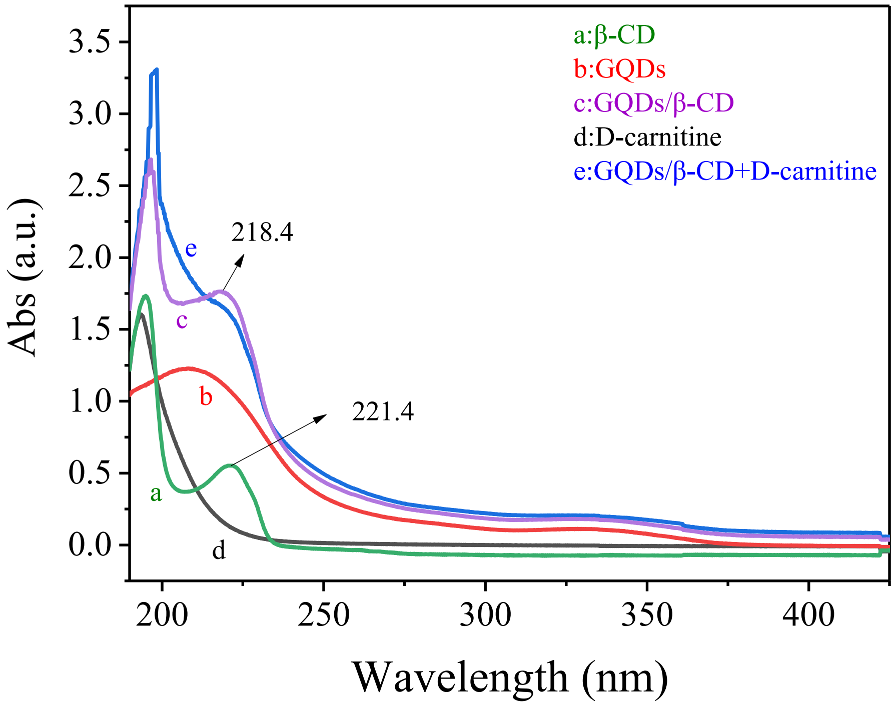

Figure S2. UV absorption spectra of, β-CD, GQDs, GQDs/β-CD, D-carnitine and (GQDs/β-CD)+D-carnitine.

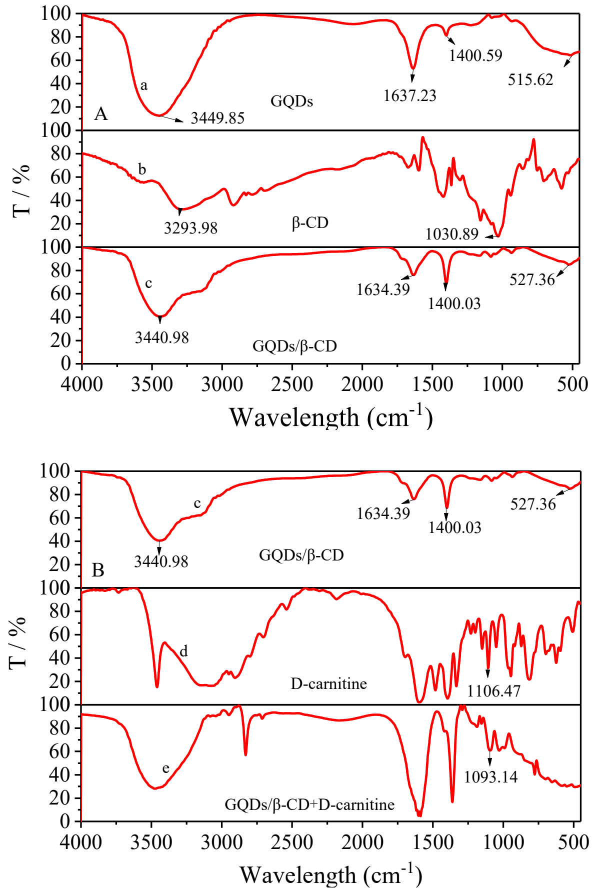

Figure S3. FT-IR sepectra of (A) GQDs, β-CD and GQDs/β-CD; and (B) GQDs/β-CD+D-carnitine, GQDs/β-CD and D-carnitine.

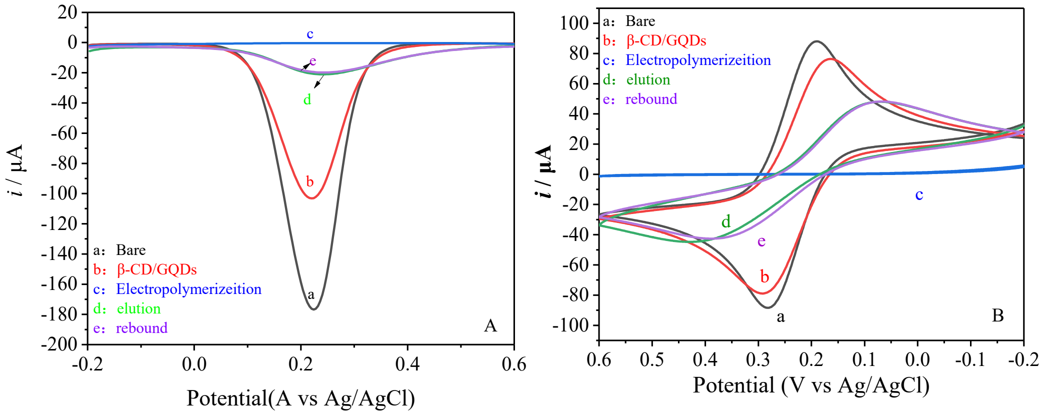

Figure S4. DPV(C) and CV(D) responses of NIPs/(GQDs/*β*-CD) sensor under different conditions. a: bare GCE, b: modified GQDs/*β*-CD, c: electropolymerization, d:elution, e: rebound

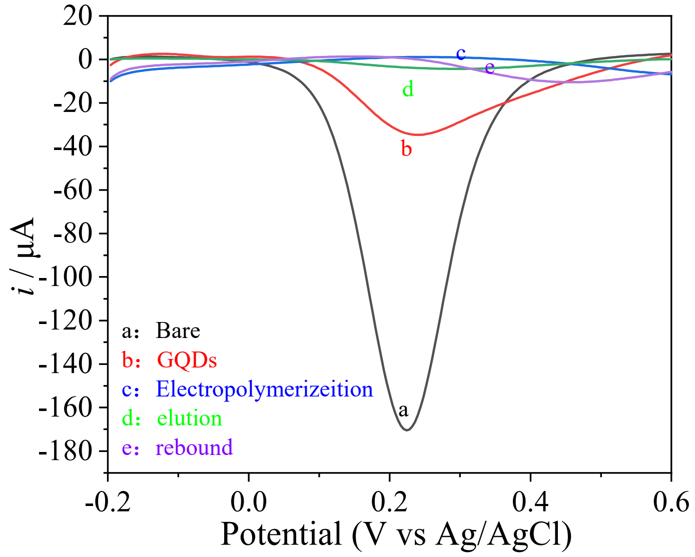

Figure S5. DPV responses of MIPs/GQDs sensor under different conditions. a: bare GCE, b: modified GQDs/β-CD, c: electropolymerization, d:elution, e: rebound

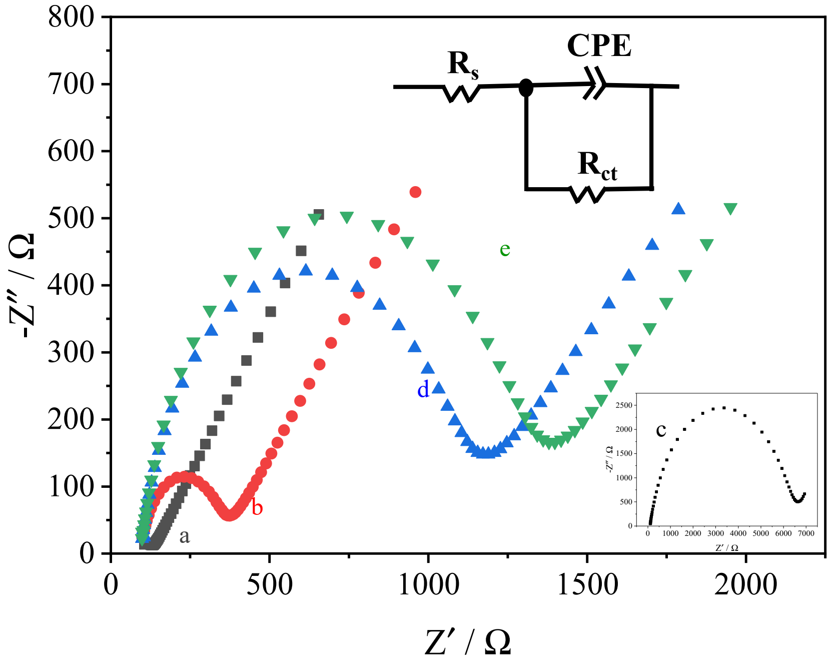

Figure S6. EIS response of MIPs/(GQDs/β-CD) sensor. a: bare GCE, b: modified GQDs/β-CD, c: electropolymerization, d: elution, e: rebound; and the insert is equivalent circuit.

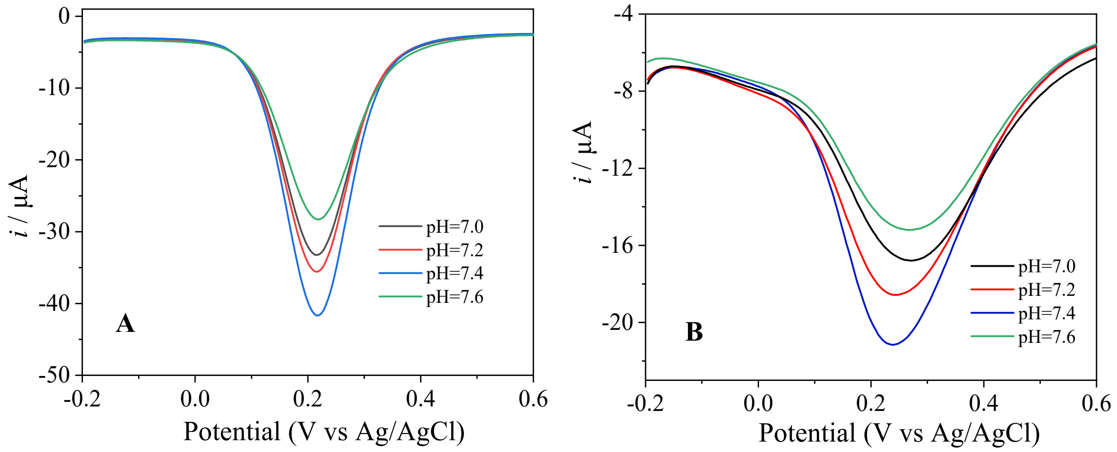

Figure S7. DPV curves of MIPs/(GQDs/β-CD) sensor under the conditions of (A) elution and (B) rebound at the different pH values of PBS.

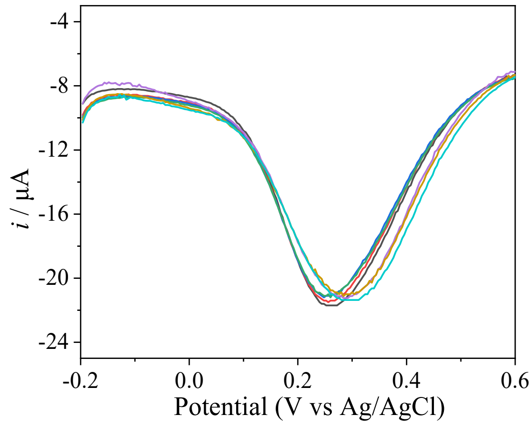

Figure S8. DPV signal response of the sensor to different concentrations of L-carnitine . (1.0×10^{-12} mol/L, 5.0×10^{-12} mol/L, 1.0×10^{-11} mol/L, 5.0×10^{-11} mol/L, 1.0×10^{-10} mol/L, 5.0×10^{-10} mol/L, 1.0×10^{-9} mol/L of L-carnitine).

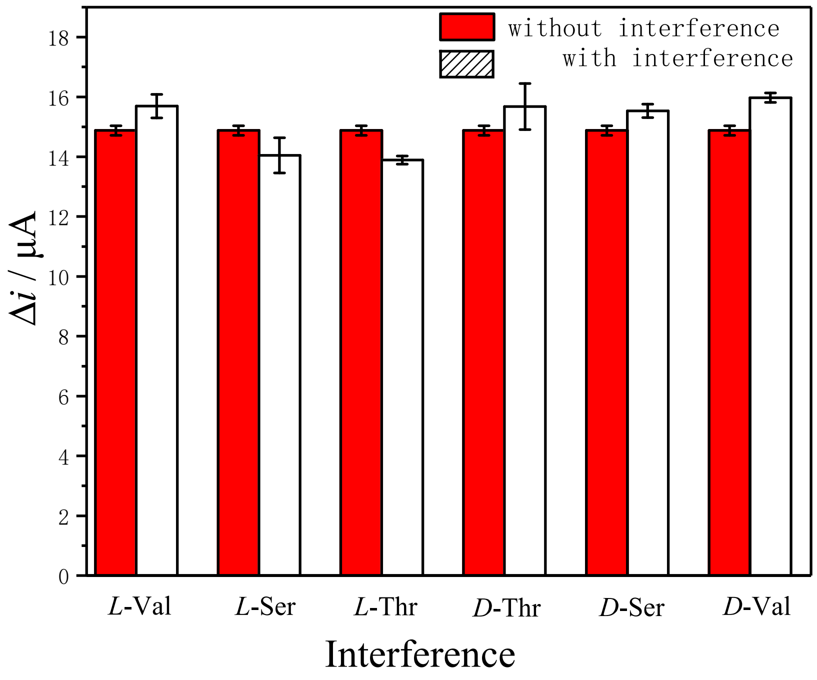

Figure S9. Anti-interference experiment of MIPs/(GQDs/β-CD) sensor against D-Thr, D-Ser, D-Val, L-Thr, L-Ser, L-Val at 1000 times concentration.

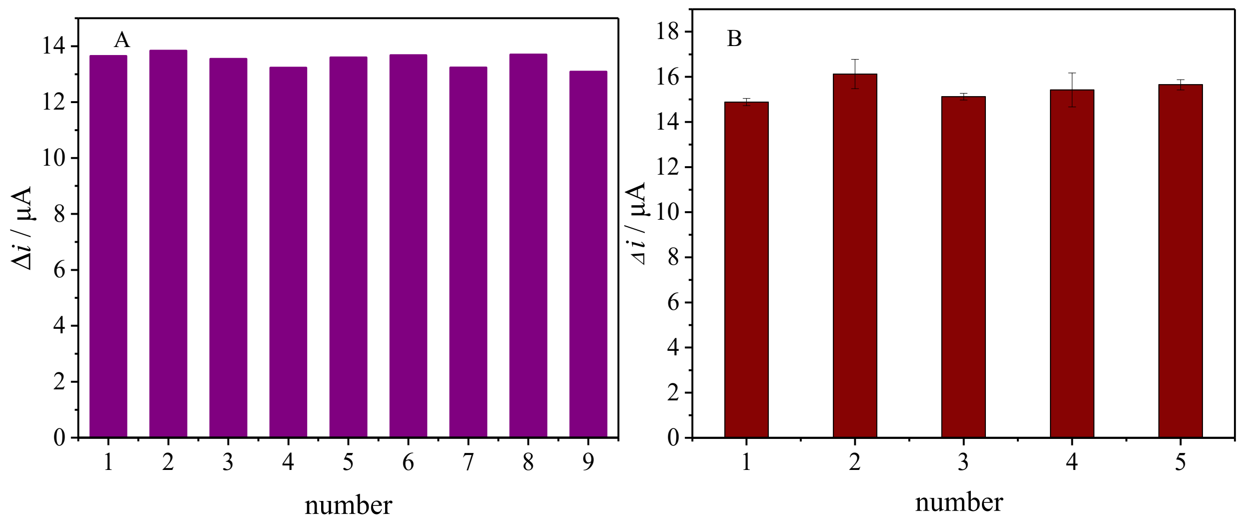

Figure S10. Determination of (A) stability and (B) reproducibility of the MIPs/(GQDs/β-CD) sensor
